# Supplementary material for: Moderate Aerobic Exercise Induces Homeostatic IgA Generation in Senile Mice
Source: Int J Mol Sci. 2024 Jul 27;25(15):8200. doi: 10.3390/ijms25158200 (PMC11311420; doi:10.3390/ijms25158200)
Supplement: Supplementary file 1 [file ijms-25-08200-s001.zip › Supplementary Figure Legends.pdf]

Supplementary Figure Legends

Supplementary Figure S1. Representative dot-plots of B cells subpopulations in lamina propria of the small intestine of sedentary and exercised mice at different ages. (**a**) total B cells (CD19+/B220+); (**b**) membrane IgM+/IgD+ B cells; (**c**) membrane IgA+ B cells.

Supplementary Figure S2. Representative dot-plots of plasma cells subpopulations in lamina propria of the small intestine of sedentary and exercised mice at different ages. (**a**) plasma cells (CD138+ cells); (**b**) cytoplasmic IgM+ plasma cells (IgM+/IgA- cells) and cytoplasmic IgA+ plasma cells (IgM-/IgA+ cells).
