# Supplementary figures and images for: Moderate Aerobic Exercise Induces Homeostatic IgA Generation in Senile Mice
Source: Int J Mol Sci. 2024 Jul 27;25(15):8200. doi: 10.3390/ijms25158200 (PMC11311420; doi:10.3390/ijms25158200)

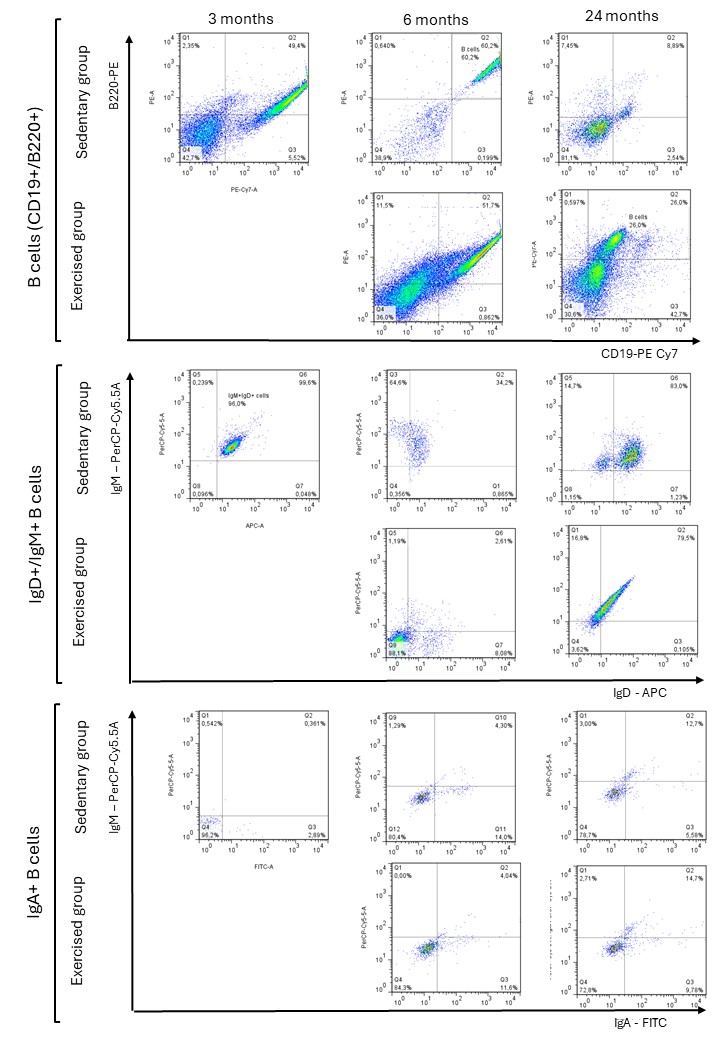

Supplement: Supplementary file 1 [file ijms-25-08200-s001.zip › SUPPL FIGURE S1.jpg]

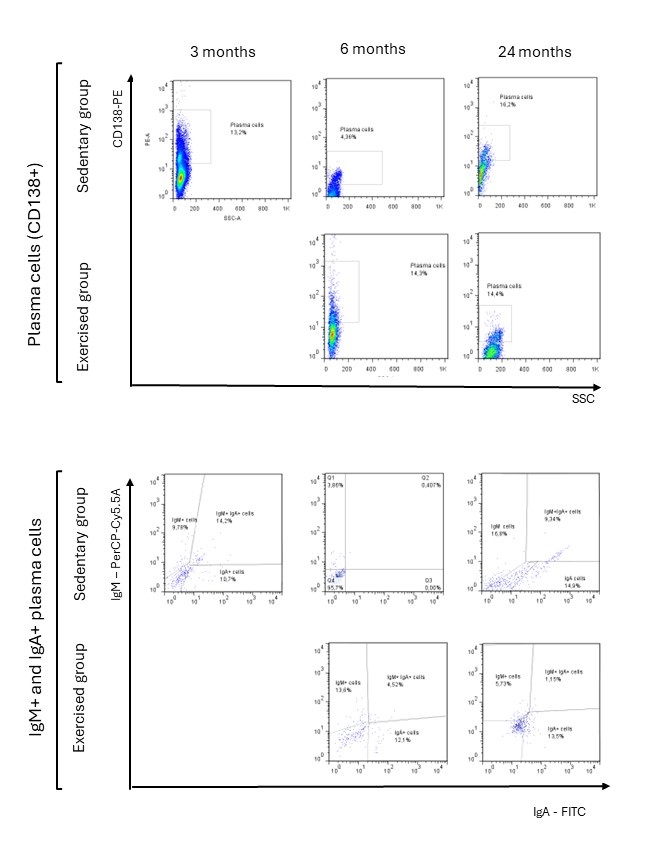

Supplement: Supplementary file 1 [file ijms-25-08200-s001.zip › SUPPL FIGURE S2.jpg]
